# Targeting the Glutamine Transporter SLC1A5 Enhances Sensitivity of Acute Myeloid Leukemia to MLN4924

**DOI:** 10.3390/biomedicines14030667

**Published:** 2026-03-14

**Authors:** Yin Wang, Yuancheng Guo, Xiao Tang, Yu Zhu, Haiping Liang, Yali Zhang, Bei Liu

**Affiliations:** 1 The First Clinical Medical College, Lanzhou University, Lanzhou 730000, China; wangyininin@163.com (Y.W.);; 2 Department of Hematology, The First Affiliated Hospital of Lanzhou University, Lanzhou 730000, China; 3 Gansu Provincial Clinical Medical Research Center for Molecular Diagnosis and Treatment of Hematological Diseases, Lanzhou 730000, China

**Keywords:** acute myeloid leukemia, neddylation, glutamine metabolism, GLUL, SLC1A5, MLN4924, V9302

## Abstract

**Background/Objectives**: Acute myeloid leukemia (AML) remains a hematologic malignancy with poor prognosis. The neddylation inhibitor MLN4924 has demonstrated potent anti-leukemic activity in preclinical models, yet its clinical translation faces significant challenges. The aim of this study was to explore combination therapy strategies that could further enhance MLN4924’s anti-leukemia potential. **Methods**: AML cell lines used in this study were Kasumi-1 and MOLM-13. Cell viability was assessed using CCK-8 assays. mRNA and protein expression levels were determined through RT-qPCR and Western blot, respectively. Flow cytometry was employed to analyze surface markers (SLC1A5, CD11b, CD14, CD16), mitochondrial membrane potential (JC-1), and apoptosis (Annexin V-FITC/PI). In vivo efficacy was validated using an NCG mouse xenograft model. Transcriptomic profiling was performed to explore the potential mechanism by which MLN4924 in combination with V9302 inhibits leukemia. **Results**: Treatment with MLN4924 significantly upregulated key glutamine metabolic proteins, GLUL and the glutamine transporter SLC1A5, in AML cells. Knockdown of SLC1A5 significantly enhanced AML cell sensitivity to MLN4924. The combination of MLN4924 and the SLC1A5 inhibitor V9302 synergistically inhibited AML cell proliferation, induced monocytic differentiation, and promoted apoptosis. Transcriptomic analysis revealed that this combination therapy prominently suppressed the tricarboxylic acid (TCA) cycle. **Conclusions**: Neddylation inhibition induces compensatory upregulation of glutamine metabolism in AML. Co-targeting neddylation and glutamine transporter SLC1A5 synergistically exerts anti-leukemic effects, at least in part through disruption of the TCA cycle. This combination represents a novel and effective therapeutic strategy against AML.

## 1. Introduction

Acute myeloid leukemia (AML) is a hematological malignancy with high biological heterogeneity [1]. With the continuous updating of treatment strategies, the prognosis of AML patients has improved significantly, but overall survival remains poor [1,2]. It is urgent to explore treatment strategies that can further prolong the survival of AML patients.

Neddylation is a critical post-translational modification that is essential for maintaining cellular homeostasis [3]. In AML, the neddylation process is overactivated and associated with poor prognosis, suggesting the therapeutic potential of targeted neddylation [4]. MLN4924, also known as pevonedistat, is a small molecule drug targeting the neddylation pathway [5]. Some preclinical studies have shown that MLN4924 has excellent anti-leukemia effects, and the mechanisms of action mainly involve DNA damage, cell cycle arrest, induction of apoptosis, and induction of cell differentiation [4,6,7,8,9,10,11]. The researchers focused not only on the anti-AML effects of MLN4924 alone but also on the anti-AML effects of MLN4924 in combination with other drugs. Several preclinical studies have explored the potential of MLN4924 to inhibit AML in combination with other drugs (Lenalidomide, azacitidine, venetoclax, T-3775440, belinostat, cytarabine, etc.) [12,13,14,15,16,17]. MLN4924’s good safety profile merits further exploration to potentially enable it to exert greater anti-AML potential.

To explore potential strategies to further enhance MLN4924’s anti-AML effect, we performed proteomic sequencing. We focused on key proteins and molecular pathways that were significantly dysregulated after MLN4924 treatment. Based on proteomic analysis results, we identified significant upregulation of a key enzyme in glutamine metabolism (glutamine synthetase, GLUL) after MLN4924 treatment. GLUL is a key enzyme responsible for glutamine synthesis [18]. GLUL has been shown to be significantly upregulated after glutamine deprivation [19], so we hypothesized that the upregulation of GLUL may be a signal that MLN4924 promotes glutamine metabolism, leading to a relative lack of glutamine in AML cells. Previous studies have shown that AML cell survival depends on glutamine, and targeting glutamine metabolism is a potential therapeutic strategy [20]. Therefore, we explored in depth the effect of MLN4924 on GLUL (a key enzyme involved in endogenous glutamine synthesis) and SLC1A5 (a key transporter involved in exogenous glutamine uptake) and explored whether a combined strategy targeting neddylation and glutamine metabolism could synergistically inhibit AML.

In this study, we focused on the regulation of two key glutamine metabolism-related proteins (GLUL and SLC1A5) by MLN4924 and explored the synergistic inhibitory effect of co-targeting neddylation and glutamine metabolism on AML. We hope our study will provide new ideas for future clinical applications.

## 2. Materials and Methods

### 2.1. Cell Culture and Reagents

The human AML cell lines Kasumi-1 and MOLM-13 were used in this study. Kasumi-1 was purchased from the American Type Culture Collection (ATCC, Manassas, VA, USA), and MOLM-13 was provided by Dr. Yuancheng Guo. Kasumi-1 and MOLM-13 underwent authentication through STR profiling prior to the experiment. All AML cells were cultured in RPMI-1640 medium (with 2 mM L-glutamine, Procell, Wuhan, China) or RPMI-1640 medium (without L-glutamine, Procell, China) containing 10% Fetal Bovine Serum (FBS, Cellbox, Changsha, China) at 5% CO_2_ and 37 °C. MLN4924 and APTO-253 were purchased from TargetMol (Boston, MA, USA), and V9302 and MSO were purchased from MedChemExpress (MCE, Monmouth Junction, NJ, USA).

### 2.2. Public Data Collection and Analysis

The gene expression data of the GLUL and SLC1A5 in adult AML and normal adult bone marrow tissues for this study were obtained from the BloodSpot database (https://www.fobinf.com/) [21]. And, data from the BloodSpot database and UALCAN database (https://ualcan.path.uab.edu/index.html) validated the correlation between GLUL and SLC1A5 expression levels and the survival of AML patients. The date of obtaining data from the above public databases was 1 June 2025.

### 2.3. RNA Purification and Reverse Transcription qPCR (RT-qPCR)

Total RNA was extracted from AML cell lines using the SPARKeasy RNA Purification Kit (SparkJade, China). Total RNA (1 µg) was reverse-transcribed to complementary DNA (cDNA) using the SPARKscript II All-in-one RT SuperMix for qPCR (SparkJade, Jinan, China). Quantitative polymerase chain reaction (qPCR) was conducted using SYBR Green qPCR Mix (SparkJade, China). The relative gene expression was calculated by correlating the expression of the GAPDH. The following primers (5′ to 3′) were used in the qPCR reactions: human GAPDH: forward, GCCAACGTGTCAGTGGTG; reverse, AAGGTGGAGGAGTGGGTGT; human GLUL: forward, GAGGAGATGGGGACAGG; reverse, CCACAGCCAACACAAGAA; human SLC1A5: forward, TTCCTCTTCACCCGCAA; reverse, GCCACGCCATTATTCTCC. All primers were synthesized by Sangon Biotechnology Co., Ltd. (Shanghai, China).

### 2.4. Immunoblotting

Total protein was isolated from the samples of AML cells using RIPA lysis solution containing protease and phosphatase inhibitors (Share-Bio, Shanghai, China). Equivalent quantities of protein samples were then subjected to 10% or 15% SDS-polyacrylamide gel electrophoresis and transferred to PVDF membranes (Millipore, Burlington, MA, USA). The membrane was then blocked with Protein Free Rapid Blocking Buffer (Epizyme, Shanghai, China), incubated with the primary antibody overnight at 4 °C, and incubated with the secondary antibody; then, the membrane was washed with TBST and finally visualized using an enhanced chemiluminescence (ECL) detection kit (Epizyme, China). Each PVDF membrane was incubated with the target protein first then stripped with Western Blot Fast Stripping Buffer (Epizyme, China) and incubated with the antibody again, GAPDH. The gray value of the WB band was semi-quantified using Image J 1.54k. Antibody against SLC1A5 (20350-1-AP) was purchased from Proteintech (Wuhan, China). Antibody against GLUL (R014807) and GAPDH (LF205) was purchased from Epizyme (China). Antibody against Caspase-3 (S0B0368) was purchased from STARTER-Bio (Hangzhou, China).

### 2.5. Cell Viability Assay

Cell proliferation ability was measured using the Cell Counting Kit-8 (CCK-8; Gooniebio, Guangzhou, China) assay. The cells in the logarithmic growth phase were inoculated into 96-well cell culture plates at the same density. CCK-8 solution was added to each well at the specified time points and incubated for another 2 h. The absorbance at 450 nm was measured using a microplate reader.

### 2.6. Relative Consumption of Glutamine in Culture Supernatant

Relative consumption of glutamine was measured with the Glutamine Assay Kit (BL1411B, LABGIC, Beijing, China) following the manufacturer’s instructions. Relative glutamine consumption was calculated by measuring the change in glutamine concentration in the medium before and after drug treatment. The medium routinely contains 2 mM glutamine.

### 2.7. Knockdown of GLUL in AML Cell Lines

Knockdown of GLUL in AML cells was achieved through transfection of small interfering RNA (siRNA) at a working concentration of 100 nM. Cells transfected with a non-targeting siRNA (scrambled siRNA) were the negative control in all knockdown experiments. Knockdown efficiency was assessed through RT-qPCR and Western blotting after 48 h of transfection. The siRNA was synthesized by Sangon Biotechnology Co., Ltd. (Shanghai, China).

### 2.8. Knockdown of SLC1A5 in AML Cell Lines

AML cells were infected with lentiviral particles containing negative controls or short hairpin RNA (shRNA) targeting SLC1A5 according to the manufacturer’s instructions (Shanghai Genechem Co., Ltd., Shanghai, China). Positively infected cells were selected with puromycin (Beyotime, Shanghai, China) treatment. Knockdown efficiency was assessed through RT-qPCR and Western blotting.

### 2.9. Flow Cytometric Analyses

#### 2.9.1. Surface SLC1A5 Analysis

Antibody against SLC1A5 (20350-1-AP) was purchased from Proteintech (China). Cells were stained with SLC1A5 antibody for 30 min at 4 °C, washed twice in PBS with 5% FBS, stained with the secondary antibody (Donkey anti-Rabbit IgG-APC Antibody; Absin, Shanghai, China) in the dark for 30 min at room temperature, and washed twice in PBS with 5% FBS. Acquisition of the data was performed on a flow cytometer (NovoCyte, Agilent Technologies, Santa Clara, CA, USA) and analyzed with FlowJo 10.8.1 software.

#### 2.9.2. Cell Differentiation Analysis

Cell differentiation was assessed through flow cytometry for CD11b, CD14, and CD16 expression on the cell surface. Antibody against CD11b (Anti-Human CD11b Mouse IgG2a Recombinant Antibody, FITC-65582) was purchased from Proteintech (China). Antibody against CD14 (Anti-CD14 Mouse mAb, 83F86E77) and CD16 (Anti-CD16 Mouse mAb, 52Q46N54) was obtained from Epizyme (China). Cells were stained with antibodies in the dark for 30 min at 4 °C. Acquisition of the data was performed on a flow cytometer (NovoCyte, Agilent Technologies, USA) and analyzed with FlowJo 10.8.1 software.

#### 2.9.3. Cell Cycle Analysis

Cell cycle analysis was performed using the cell cycle detection kit (Epizyme, China). AML cells were fixed with 70% ethanol overnight at −20 °C, followed by incubating with RNase A for 15 min at 37 °C, then stained with propidium iodide (PI) in the dark for 10 min at 4 °C. Acquisition of the data was performed on a flow cytometer (NovoCyte, Agilent Technologies, USA) and analyzed with FlowJo 10.8.1 software.

#### 2.9.4. Cell Apoptosis Analysis

To assess apoptosis, an Annexin V-FITC/PI apoptosis analysis kit (Epizyme, China) was used in the AML cell lines (Kasumi-1 and MOLM-13). AML cells incubated with Annexin V-FITC and PI for 10 min to detect apoptosis. Acquisition of the data was performed on a flow cytometer (NovoCyte, Agilent Technologies, USA) and analyzed with FlowJo 10.8.1 software.

#### 2.9.5. Mitochondrial Membrane Potential (MMP) Assay

To examine the changes in the mitochondrial membrane potential (MMP), AML cells were stained with the JC-1 dye kit (Solarbio, Beijing, China), and acquisition of the data was performed on a flow cytometer (NovoCyte, Agilent Technologies, USA). The change from aggregates to monomers of JC-1 based on fluorescence intensity was analyzed with FlowJo 10.8.1 software.

### 2.10. Cell Morphology Analysis

Kasumi-1 and MOLM-13 cells were treated with MLN4924 and V9302 for 24 h, and then the cells were collected. Slides were made and then air-dried. The cells were stained with Wright-Giemsa, and their morphological characteristics were observed with an optical microscope (Nikon, Tokyo, Japan).

### 2.11. Astral-DIA Proteomics

Kasumi-1 cells were treated with MLN4924 or DMSO for 24 h before protein profiling. Astral-DIA proteomics was conducted by Hangzhou Cosmos Wisdom Biotech Co., Ltd. (Hangzhou, China). The analysis was performed using the nanoflow rate Vanquish Neo system (Thermo Scientific, Waltham, MA, USA) for chromatographic separation and the Astral high-resolution mass spectrometer (Thermo Scientific) for Data Independent Acquisition (DIA) mass spectrometry analysis of samples separated through high-efficiency liquid chromatography. The detection mode was positive ion, with a precursor ion scan range of 380–980 *m*/*z* and a primary mass spectrometry resolution of 240,000 at 200 *m*/*z*. Normalized AGC Target was set at 500%, with a Maximum IT of 5 ms. MS2 employed the DIA data acquisition mode, with 299 scan windows set, an Isolation Window of 2 *m*/*z*, HCD Collision Energy set at 25 ev, Normalized AGC Target at 500%, and a Maximum IT of 3 ms. After processing the raw data, the available and valid data were obtained, and then data mining was carried out.

### 2.12. RNA-Seq and Data Analysis

RNA-seq was conducted by Hangzhou Cosmos Wisdom Biotech Co., Ltd. (Hangzhou, China). Total RNA was extracted, and RNA quality was determined using the 2100 Bioanalyser and quantified using the ND-2000 (NanoDrop Technologies, Wilmington, DE, USA). A high-quality RNA sample was used to construct sequencing libraries. RNA-seq transcriptome libraries were prepared following the Illumina TruseqTM RNA sample prep kit (San Diego, CA, USA). Shortly, messenger RNA was isolated with polyA selection by oligo (dT) beads and fragmented using fragmentation buffer. cDNA synthesis, end repair, A-base addition, and ligation of the Illumina-indexed adaptors were performed. Libraries were then size selected for cDNA target fragments on 2% Low Range Ultra Agarose followed by PCR amplified using Phusion DNA polymerase (NEB) for 15 PCR cycles. After quantification by TBS380, paired-end libraries were sequenced through Illumina NovaSeq 6000 sequencing (150 bp*2). After processing the raw data, the available data were obtained, and then gene expression analysis and other data mining were carried out.

### 2.13. AML Cell-Line-Derived Xenograft (CDX) Models

All animal experiments were conducted at the animal facility of Lanzhou University (Lanzhou, China). Each protocol applied was approved by the Ethics Committee of the First Affiliated Hospital of Lanzhou University (Ethics Lot number: LDYYLL-2025-2016), and investigators followed the ethical code of animal use. In this study, NCG mice (female, 6 weeks old) (Cat. NO. T001475) were purchased from GemPharmatech Company (Chengdu, China). Animals were allowed to acclimate to the environment for 7 days in a specific-pathogen-free (SPF) room. After 7 days of acclimatization, NCG mice were randomly grouped. MOLM-13 cells (2 × 10^6^) were injected subcutaneously into the right flanks of NCG mice. One week after inoculation of tumor cells, treatment was given as planned. The control group received intraperitoneal injection of the vehicle, the MLN4924 group received MLN4924 60 mg/kg once daily intraperitoneally, the V9302 group received V9302 20 mg/kg once daily intraperitoneally, and the combination group received both MLN4924 and V9302 once daily intraperitoneally. Tumor volume and body weight of all mice were monitored once every three days, and some general states, such as activity ability and hair, were observed every day. Tumor volumes were calculated using the formula Tumor Volume (mm^3^) = 0.5 × (a × b^2^), where a is the long diameter of the tumor and b is the short diameter. If the subcutaneous tumor volume of different groups of mice was significantly different (the experimental purpose was achieved), the experiment was terminated considering animal welfare. In addition, if the animal showed significant weight loss, poor mobility, and other general poor conditions, animal welfare was considered to terminate the experiment.

### 2.14. Drug Synergy Analysis

To identify synergistic effects of the two-drug combinations, CalcuSyn software version 1.0 was used to calculate the Combination Index (CI). A CI value > 1 indicates antagonism; a CI value < 1, synergy; and a CI value = 1, additive effect.

### 2.15. Statistical Analysis

All statistical comparisons were evaluated using Student’s *t* test or one-way or two-way analysis of variance (ANOVA). For Kaplan–Meier survival analysis, the log-rank test was employed for comparison of two groups. Data was analyzed using GraphPad Prism version 9.0. * *p* < 0.05, ** *p* < 0.01, *** *p* < 0.001, **** *p* < 0.0001; ns: not significant.

## 3. Results

### 3.1. Protein Profiling Reveals Potential Effects of MLN4924 Treatment on Glutamine Metabolism in AML

**Figure 1:**
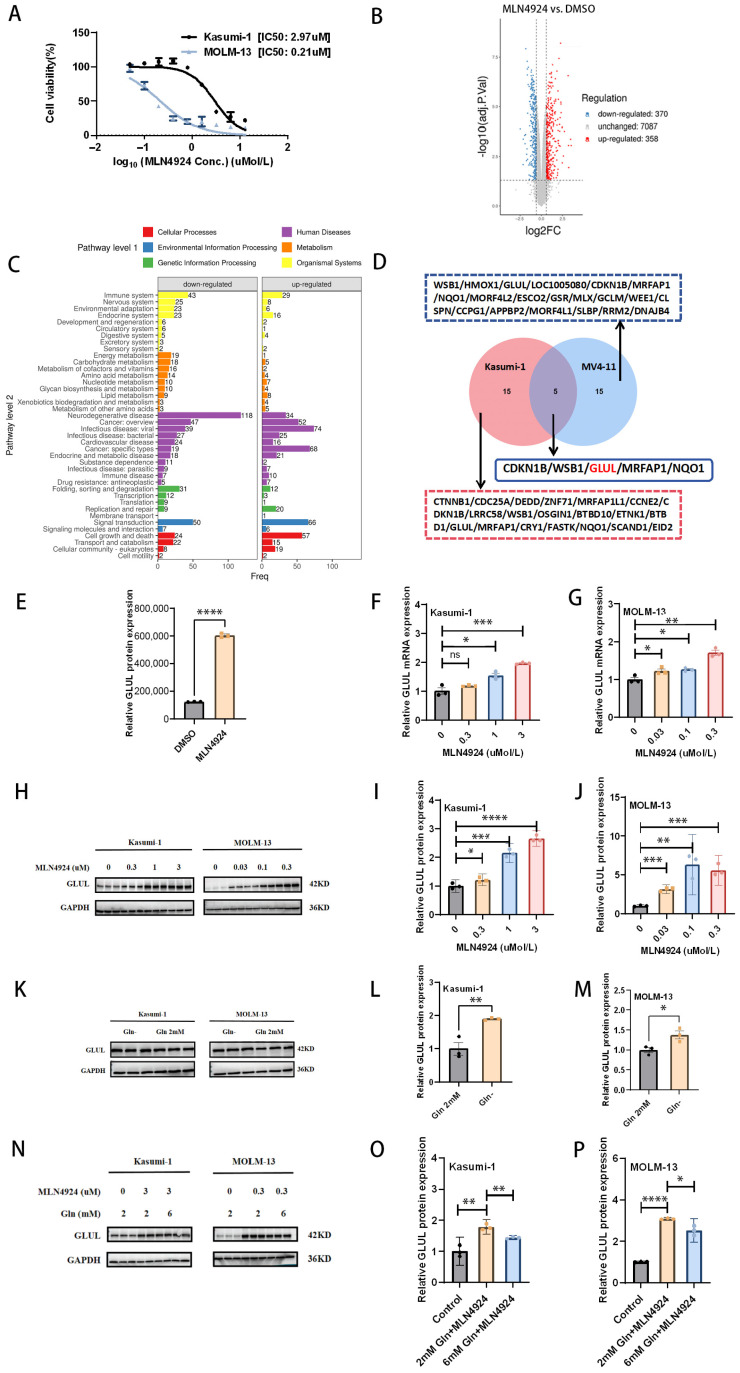
Protein profiling reveals potential effects of MLN4924 treatment on glutamine metabolism in AML. (**A**) Kasumi-1 and MOLM-13 cells were treated with MLN4924 for 24 h and IC50 was detected through CCK8 assay. (**B**) Kasumi-1 cells were treated with MLN4924 and DMSO for 24 h and then protein profiling was performed. Volcano plot depicts differential proteins between DMSO and MLN4924-treated groups, with red indicating upregulated and blue indicating downregulated proteins (MLN4924 vs. DMSO). (**C**) KEGG enrichment analysis visualized the top pathways (MLN4924 vs. control). (**D**) Venn diagram illustrates the overlapped targets of MLN4924 vs. control in Kasumi-1 and MV4-11. (**E**) Proteomic analysis revealed differences in GLUL between DMSO and MLN4924-treated groups. (**F**,**G**) Evaluate the expression of GLUL after MLN4924 treatment for 24 h through RT-qPCR. (**H**–**J**) Western blot was used to detect the expression of GLUL protein after MLN4924 treatment for 24 h. (**K**–**M**) Western blot to verify that exogenous glutamine deprivation resulted in upregulation of GLUL expression (24 h after treatment). (**N**–**P**) Western blot to verify that exogenous glutamine supplementation reverses the upregulation of GLUL by MLN4924 (24 h after treatment). * *p* < 0.05, ** *p* < 0.01, *** *p* < 0.001, **** *p* < 0.0001; ns: not significant.

MLN4924 has been shown to inhibit AML in several studies but has not shown very surprising results in several clinical trials. To explore ways to further enhance the therapeutic effect of MLN4924, we sought to explore potential strategies through protein profiling. First, we verified that MLN4924 significantly inhibited AML cell viability with IC50 of 2.97 µM in Kasumi-1 cells and 0.21 µM in MOLM-13 cells [Figure 1A]. Kasumi-1 cells were selected for protein profiling. A total of 728 differential proteins were identified, with 358 upregulated and 370 downregulated [Figure 1B]. KEGG enrichment analysis of these differential proteins revealed that MLN4924 affected multiple metabolic pathways in AML cells [Figure 1C]. Next, we focused on the top 20 proteins that were significantly upregulated. Our goal was to find more universal potential targets, so we combined our data with those of Visconte V et al. [6]. MV4-11 was selected for protein profiling in the study by Visconte V et al. [6]. The intersection of the top 20 upregulated proteins in the two studies yielded 5 shared proteins [Figure 1D]. After reviewing many studies, we found that inhibition of GLUL has a synergistic effect on other antitumor therapies [22,23], so we regard GLUL as a key target for further research. Protein profiling suggested that GLUL was significantly upregulated after MLN4924 treatment [Figure 1E]. To further verify the protein profiling results, we verified the regulatory effect of MLN4924 on GLUL expression by RT-qPCR [Figure 1F,G] and Western blot [Figure 1H–J]. The results showed that MLN4924 treatment significantly upregulated GLUL at both mRNA and protein levels in Kasumi-1 and MOLM-13 cells.

Because previous studies have shown that GLUL is significantly upregulated in glutamine deficiency [19], we hypothesized that MLN4924 treatment upregulates GLUL due to the relative intracellular glutamine deficiency caused by the reprogramming of glutamine metabolism. The upregulation of GLUL may be a signal of the shortage of glutamine in AML cells after MLN4924 treatment. To test this hypothesis, we first examined the expression of GLUL after 24 h of glutamine deprivation, which showed an upregulation of GLUL expression compared to normal culture conditions (with 2 mM glutamine) [Figure 1K–M]. Next, we examined the expression of GLUL after exogenous glutamine supplementation, and the results showed that exogenous glutamine supplementation reversed the upregulation of GLUL caused by MLN4924 [Figure 1N–P]. These results suggest that glutamine metabolism reprogramming occurs after MLN4924 treatment and AML cells have a relatively increased demand for glutamine.

### 3.2. MLN4924 Promotes Glutamine Consumption by AML Cells and Upregulates Cell Surface SLC1A5 Expression

**Figure 2:**
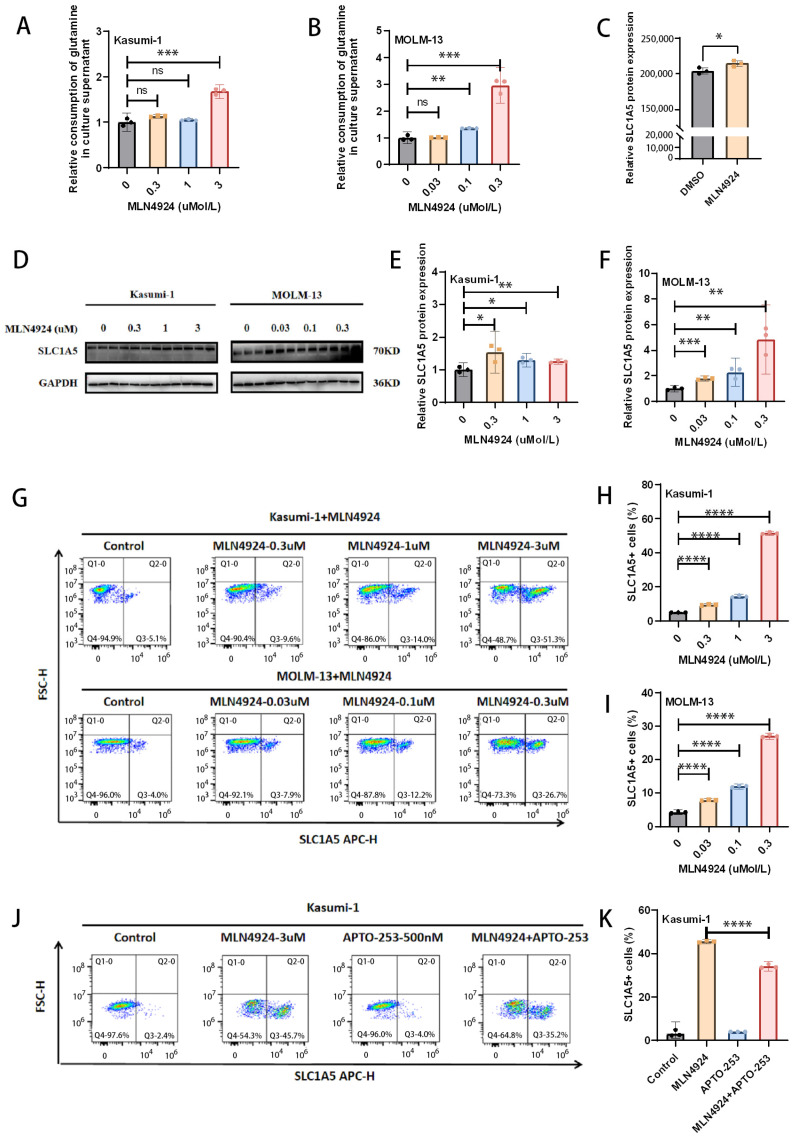
MLN4924 promotes glutamine consumption by AML cells and upregulates cell surface SLC1A5 expression. (**A**,**B**) Relative consumption of glutamine in culture supernatant by AML cells was evaluated by detecting the change in glutamine content in culture medium (24 h after treatment). (**C**) Proteomic analysis revealed differences in SLC1A5 between DMSO and MLN4924-treated groups (24 h after treatment). (**D**–**F**) Western blot was used to detect the expression of SLC1A5 protein after MLN4924 treatment for 48 h. (**G**–**I**) The expression of SLC1A5 on AML cell surface after MLN4924 treatment for 48 h was detected through flow cytometry. (**J**,**K**) The expression of SLC1A5 on AML cell surface after MLN4924 and APTO-253 (MYC inhibitor) treatment for 48 h was detected through flow cytometry. * *p* < 0.05, ** *p* < 0.01, *** *p* < 0.001, **** *p* < 0.0001; ns: not significant.

To further verify the effect of MLN4924 on glutamine metabolism in AML cells, we examined the effect of MLN4924 treatment on glutamine uptake by AML cells. The results showed that MLN4924 treatment increased the consumption of glutamine in culture medium [Figure 2A,B]. Glutamine is important for AML cell survival. MLN4924 treatment leads to a further increase in glutamine consumption in AML cells, suggesting that restriction of exogenous glutamine may further enhance MLN4924 inhibition of AML cells. Therefore, we focused on the regulation of key glutamine transporters SLC1A5 by MLN4924. Protein profiling showed mild upregulation of SLC1A5 after MLN4924 treatment [Figure 2C]. To further verify the protein profiling results, we verified the regulatory effect of MLN4924 on SLC1A5 expression through Western blotting. The results showed that MLN4924 treatment significantly upregulated SLC1A5 in Kasumi-1 and MOLM-13 cells [Figure 2D–F]. And, flow cytometry showed that SLC1A5 on the AML cell surface was significantly upregulated after MLN4924 treatment in a concentration-dependent manner [Figure 2G–I]. Furthermore, MYC has been found to directly activate SLC1A5 transcription [24], while APTO-253 can inhibit MYC expression [25]. Based on these results, we explored whether APTO-253 can reverse MLN4924-induced SLC1A5 upregulation by inhibiting MYC expression. The results showed that SLC1A5 expression on the cell surface decreased in the APTO-253 + MLN4924 group compared with MLN4924 alone [Figure 2J,K]. These results suggest that MLN4924 upregulates SLC1A5 expression on the cell surface and inhibition of MYC partially reverses MLN4924-induced SLC1A5 upregulation.

### 3.3. The Expression of GLUL and SLC1A5 in AML and Its Relationship with the Prognosis of AML Patients

**Figure 3:**
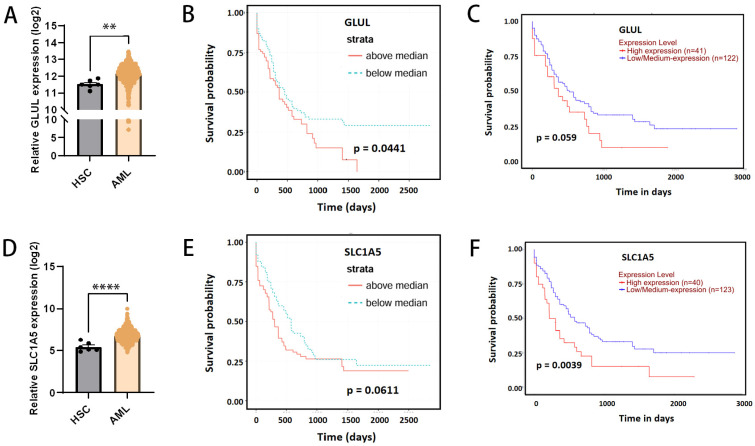
The expression of GLUL and SLC1A5 in AML and its relationship with the prognosis of AML patients. (**A**) The expression of GLUL in AML patients and healthy controls (data from BloodSpot). (**B**) The correlation between GLUL expression and prognosis of AML patients (data from BloodSpot). (**C**) The correlation between GLUL expression and prognosis of AML patients (data from UALCAN). (**D**) The expression of SLC1A5 in AML patients and healthy controls (data from BloodSpot). (**E**) The correlation between SLC1A5 expression and prognosis of AML patients (data from BloodSpot). (**F**) The correlation between SLC1A5 expression and prognosis of AML patients (data from UALCAN). ** *p* < 0.01, **** *p* < 0.0001.

Two key proteins in glutamine metabolism, GLUL (mainly responsible for endogenous glutamine synthesis) and SLC1A5 (mainly responsible for exogenous glutamine uptake), were identified in the above experiments. To further verify their expression in AML patients and the relationship between their expression levels and prognosis of the patients, we obtained gene expression information and survival information of AML patients and healthy donors through BloodSpot, a public database. Gene expression analysis showed that both GLUL and SLC1A5 were significantly upregulated in AML patients compared with hematopoietic stem cells from healthy donors [Figure 3A,D]. Survival analysis results showed that AML patients with high expression of GLUL had a significantly worse prognosis [Figure 3B,C] and, similarly, AML patients with high expression of SLC1A5 tended to have a worse prognosis [Figure 3D,E]. These results suggest that GLUL and SLC1A5 are associated with survival outcomes in AML patients.

### 3.4. Extracellular Glutamine Affects AML Cell Growth and Survival, Whereas Inhibition of GLUL Does Not Further Enhance the Anti-Leukemic Effects of MLN4924

**Figure 4:**
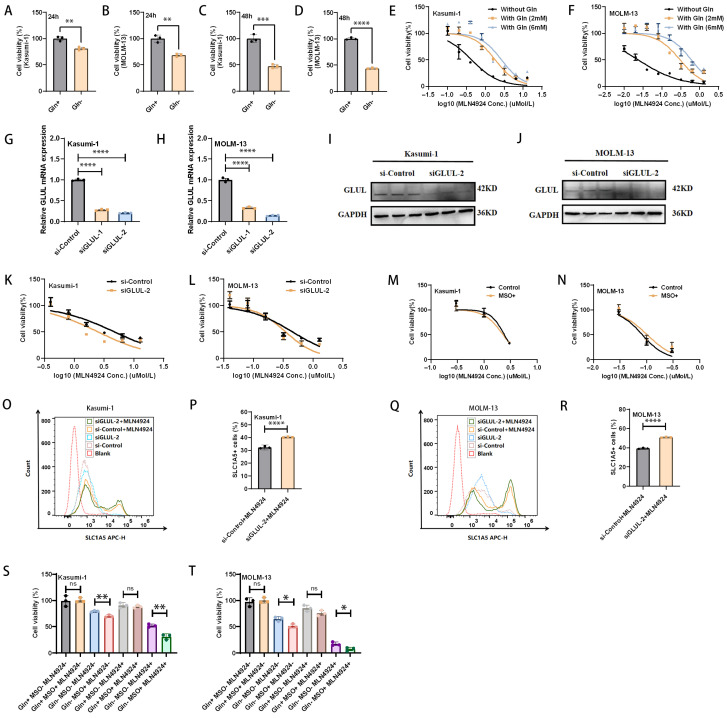
Extracellular glutamine (Gln) affects AML cell survival, whereas inhibition of GLUL does not further enhance the anti-leukemic effects of MLN4924. (**A**–**D**) CCK8 assay was used to detect the effect of exogenous glutamine removal on the cell viability of AML cells. (**E**,**F**) The effect of exogenous glutamine on the sensitivity to MLN4924 of AML cells was detected through CCK8 assay (24 h after treatment). (**G**–**J**) Evaluate the interference effect of siRNA on GLUL in AML cells through RT-qPCR and Western blot. (**K**,**L**) The effect of GLUL knockdown on the sensitivity to MLN4924 of AML cells was detected through CCK8 assay (24 h after treatment). (**M**,**N**) The effect of MSO (GLUL inhibitor) on the sensitivity to MLN4924 of AML cells was detected through CCK8 assay (24 h after treatment). (**O**–**R**) The expression of SLC1A5 on AML cell surface after GLUL knockdown was detected through flow cytometry. (**S**,**T**) CCK8 assay was used to detect the effect of GLUL inhibitor MSO (1 mM) on the sensitivity to MLN4924 (Kasumi-1: 1 μM, MOLM-13: 0.1 μM) of AML cells under glutamine (Gln) deprivation (24 h after treatment). * *p* < 0.05, ** *p* < 0.01, *** *p* < 0.001, **** *p* < 0.0001; ns: not significant.

The above results demonstrate the importance of glutamine for AML cell survival and the further increase in glutamine demand induced by MLN4924 treatment. Next, we explored the effects of exogenous glutamine deprivation on AML cell viability and AML cell sensitivity to MLN4924. The results showed that AML cell viability was significantly inhibited after 24 h and 48 h of glutamine deprivation in medium [Figure 4A–D]. And, the inhibitory effect of MLN4924 on AML cells was significantly enhanced when glutamine was deprived of culture medium, whereas the inhibitory effect of MLN4924 on AML cells was weakened when glutamine concentration was increased [Figure 4E,F]. These results demonstrate the importance of exogenous glutamine for AML cell survival.

We further examined the effect of the endogenous glutamine pathway on AML cell survival. GLUL is the main endogenous glutamine synthesis enzyme. We verified the effect of the GLUL pathway on the anti-leukemia effect of MLN4924. The results showed that neither knockdown of the GLUL nor treatment with the GLUL inhibitor MSO (1 mM) further increased the inhibitory effect of MLN4924 on AML cells [Figure 4G–N].

The inhibition of GLUL does not increase the anti-leukemic effect of MLN4924, possibly because inhibition of the endogenous glutamine synthesis pathway can be compensated by the exogenous glutamine pathway. Based on these hypotheses, we further examined whether GLUL knockdown further promotes the upregulation of SLC1A5 on AML cell surfaces by MLN4924. Interestingly, in Kasumi-1 and MOLM-13 cells, SLC1A5 was further upregulated in the siGLUL + MLN4924 group compared to the MLN4924 group [Figure 4O–R]. These results suggest that the potential mechanism of inhibition of GLUL failing to further enhance the anti-leukemia effect of MLN4924 may be compensation of the exogenous glutamine pathway, such as SLC1A5 upregulation. The specific mechanism needs to be further clarified in the future.

These results suggest that inhibition of GLUL does not enhance AML cell sensitivity to MLN4924 in the presence of glutamine. Furthermore, we validated the effect of GLUL inhibition on MLN4924 inhibition of AML in the context of glutamine deprivation. The results showed that the GLUL inhibitor MSO inhibited AML cell viability and further enhanced the anti-AML effect of MLN4924 when glutamine was withdrawn [Figure 4S,T].

### 3.5. Effect of MLN4924 in Combination with SLC1A5 Inhibitor (V9302) on AML Cell Proliferation and Cell Differentiation

**Figure 5:**
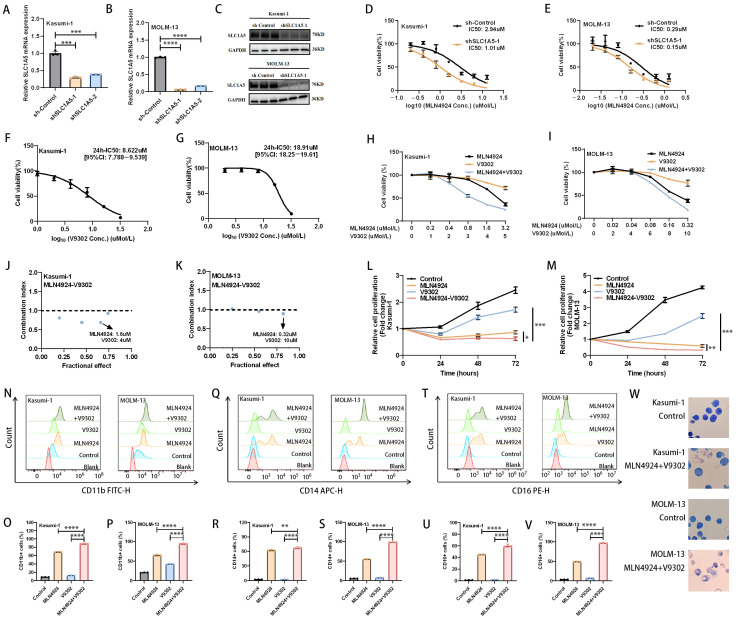
Effect of MLN4924 in combination with SLC1A5 inhibitor (V9302) on AML cell proliferation and cell differentiation. (**A**–**C**) Evaluate the interference effect of shRNA on SLC1A5 in AML cells through RT-qPCR and Western blot. (**D**,**E**) The effect of SLC1A5 knockdown on the sensitivity to MLN4924 of AML cells was detected through CCK8 assay (24 h after treatment). (**F**,**G**) Kasumi-1 and MOLM-13 cells were treated with V9302 for 24 h and IC50 was detected through CCK8 assay. (**H**–**M**) CCK8 assay was used to verify the effect of MLN4924 combined with V9302 on AML cell viability. (**N**–**V**) CD11b, CD14, and CD16 expression on AML cell surface treated with MLN4924 and V9302 for 48 h was detected through flow cytometry. (**W**) Wright–Giemsa staining to detect morphological changes of AML cells treated with MLN4924 and V9302 for 24 h captured using an oil immersion lens (1000×). * *p* < 0.05, ** *p* < 0.01, *** *p* < 0.001, **** *p* < 0.0001.

Based on the above results, we next focused on SLC1A5, a key transporter involved in exogenous glutamine uptake. First, we found that knockdown of SLC1A5 further enhanced the anti-leukemic effect of MLN4924 [Figure 5A–E]. On this basis, we investigated the effect of SLC1A5 inhibitor V9302 on AML cells. The results showed that V9302 significantly inhibits the viability of AML cells [Figure 5F,G]. Kasumi-1 cells were more sensitive to V9302 than MOLM-13 cells, with IC50 (24 h) of 8.62 µM and 18.91 µM [Figure 5F,G]. We hypothesized that V9302 may have synergistic anti-AML effects with MLN4924. Because MLN4924 has been shown to have a good safety profile in clinical studies and V9302 has not been tested in clinical trials, MLN4924 is the dominant drug in the combination. We set most concentrations of MLN4924 below IC50 and concentrations of V9302 below IC30. The results showed that MLN4924 and V9302 had synergistic anti-AML effects in Kasumi-1 and MOLM-13 cells [Figure 5H–M]. Based on the Combination Index (CI), we selected the drug concentrations with the greatest synergistic efficacy for the next study [Figure 5J,K]. In Kasumi-1, we selected the drug concentration of MLN4924 at 1.6 µM and V9302 at 4 µM [Figure 5J]. In MOLM-13, we selected the drug concentration of MLN4924 at 0.32 µM and V9302 at 10 µM [Figure 5K].

Cell differentiation disorder is one of the typical features of AML, so we evaluated the effect of MLN4924 in combination with V9302 on AML cell differentiation status. CD11b, CD14, and CD16 are markers of cell differentiation [26,27]. The results showed that MLN4924 monotherapy significantly upregulated CD11b, CD14, and CD16 expression on the cell surface in Kasumi-1 and MOLM-13 cells [Figure 5N–V]. And, surprisingly, MLN4924 in combination with V9302 further upregulated CD11b, CD14, and CD16 expression on AML cell surface [Figure 5N–V]. In addition, we further assessed cell differentiation by observing morphological changes in AML cells treated with MLN4924 and V9302. Morphology also shows differentiation [Figure 5W]. These results suggest that MLN4924 in combination with V9302 synergistically induces AML cell differentiation.

### 3.6. Combination of MLN4924 and V9302 Induces Cell Cycle Arrest, Loss of Mitochondrial Membrane Potential (MMP), and Apoptosis of AML Cells

**Figure 6:**
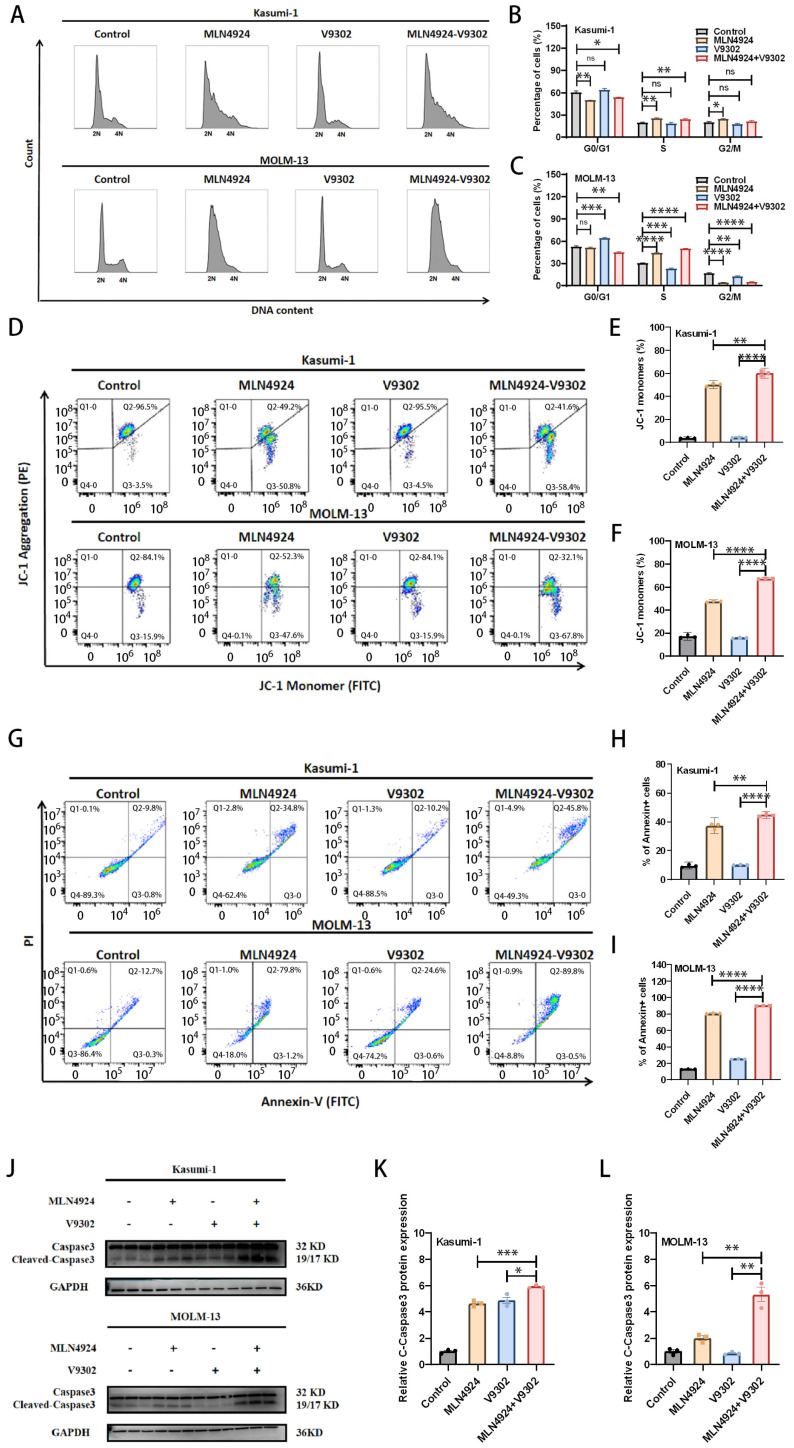
Combination of MLN4924 and V9302 induces cell cycle arrest, loss of mitochondrial membrane potential (MMP), and apoptosis of AML cells. (**A**–**C**) Cell cycle profiles were analyzed through flow cytometry (24 h after treatment). (**D**–**F**) The mitochondrial membrane potential was analyzed through flow cytometry after staining with JC-1 dye (24 h after treatment). (**G**–**I**) Apoptosis was quantified through flow cytometry using Annexin V-FITC/PI staining (24 h after treatment). (**J**–**L**) Western blotting was used to detect the expression of apoptosis-related proteins Caspase-3 in AML cells (12 h after treatment). * *p* < 0.05, ** *p* < 0.01, *** *p* < 0.001, **** *p* < 0.0001; ns: not significant.

The cell cycle is related to cell proliferation and apoptosis. We evaluated the effect of MLN4924 in combination with V9302 on the AML cell cycle. The results showed that the cells were mainly arrested in the S phase after MLN4924 treatment and the cells were mainly arrested in the G0/G1 phase after V9302 treatment [Figure 6A–C]. The cells were mainly arrested in the S phase after MLN4924 combined with V9302 treatment [Figure 6A–C]. These results suggest that MLN4924 and V9302 treatment can cause cell cycle disorders.

The stability of mitochondrial membrane potential (MMP) is an important factor in maintaining cell survival, and the change of MMP can reflect the state of cells. JC-1 staining was used to detect the effect of MLN4924 and V9302 on MMP in AML cells. The results showed that the proportion of JC-1 monomer in the MLN4924 + V9302 group was significantly higher than that in MLN4924 alone or the V9302 group alone [Figure 6D–F], suggesting that a combination of MLN4924 and V9302 significantly induced mitochondrial dysfunction in AML cells.

To assess drug-induced apoptosis, AML cells were stained with Annexin V-FITC/PI and analyzed through flow cytometry. The results showed that the proportion of apoptotic cells in the combination treatment group of MLN4924 and V9302 was higher than that in MLN4924 alone or the V9302 group alone [Figure 6G–I].

In addition, we verified the expression of apoptosis-related proteins Caspase-3 through Western blotting. The results showed that MLN4924 and V9302 co-treatment resulted in significant upregulation of cleaved Caspase-3 [Figure 6J–L].

These results suggest that MLN4924 in combination with V9302 synergistically induces apoptosis in AML cells and the mechanism involves the mitochondrial pathway.

### 3.7. Combination of MLN4924 and V9302 Synergistically Inhibits AML In Vivo

**Figure 7:**
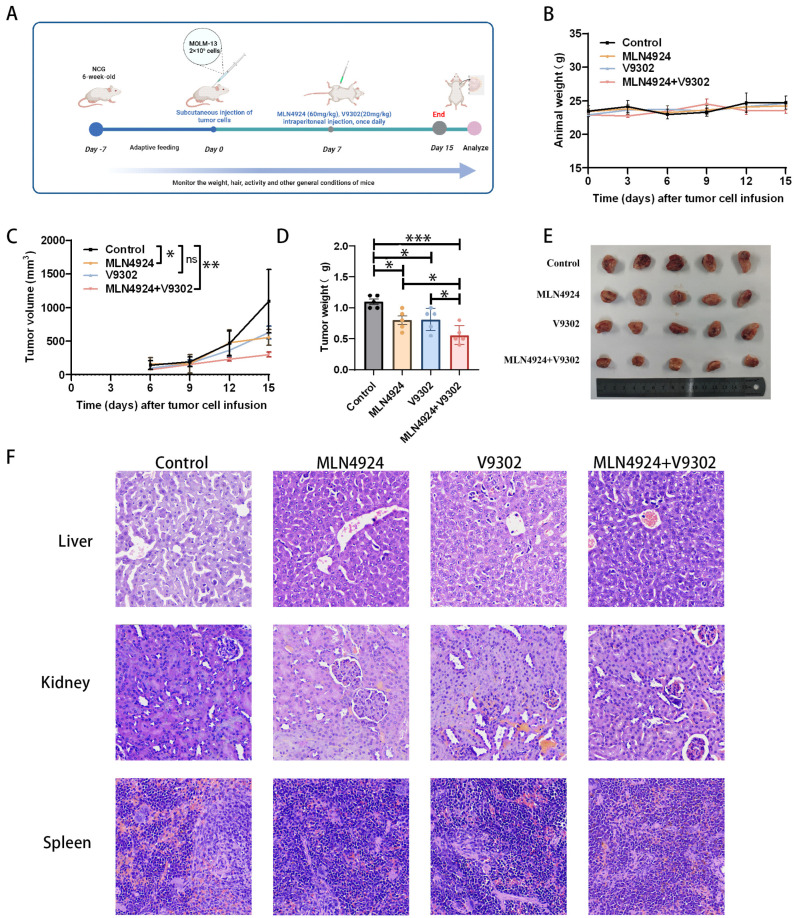
Combination of MLN4924 and V9302 synergistically inhibits AML in vivo. (**A**) In NCG mice, MOLM-13 cells were injected subcutaneously. One week after inoculation of tumor cells, treatment was given. The control group received intraperitoneal injection of vehicle, the MLN4924 group received MLN4924 60 mg/kg once daily intraperitoneally, V9302 received V9302 20 mg/kg once daily intraperitoneally, and the combination group received both drugs once daily intraperitoneally. (**B**–**E**) Tumor volume and body weight of all mice were monitored once every three days. On day 15 of cell injection, the mice were euthanized, and the tumors were removed and weighed. (**F**) Images of HE staining of liver, spleen, and kidney at the end of the experiment (200×). * *p* < 0.05, ** *p* < 0.01, *** *p* < 0.001, ns: not significant.

To validate the inhibitory ability of MLN4924 in combination with V9302 against leukemia cells in an in vivo context, we established a xenograft model [Figure 7A]. The results demonstrated that the MLN4924 monotherapy group, the V9302 monotherapy group, and the MLN4924 + V9302 group all showed weaker proliferative capacity in vivo compared with the control group, and the MLN4924 + V9302 group showed weaker proliferation than the monotherapy group [Figure 7C–E]. By the end of the experiment, both tumor volume and weight in the MLN4924 + V9302 group had significantly decreased [Figure 7C–E]. In addition, to assess the safety of MLN4924 and V9302, HE staining was performed on the liver, spleen, and kidney of mice, and no obvious pathological changes were found [Figure 7F]. Collectively, these findings indicate that the combination of MLN4924 and V9302 significantly suppressed the growth of AML cells in vivo.

### 3.8. Combination of MLN4924 and V9302 Inhibits the Tricarboxylic Acid Cycle (TCA) Cycle

**Figure 8:**
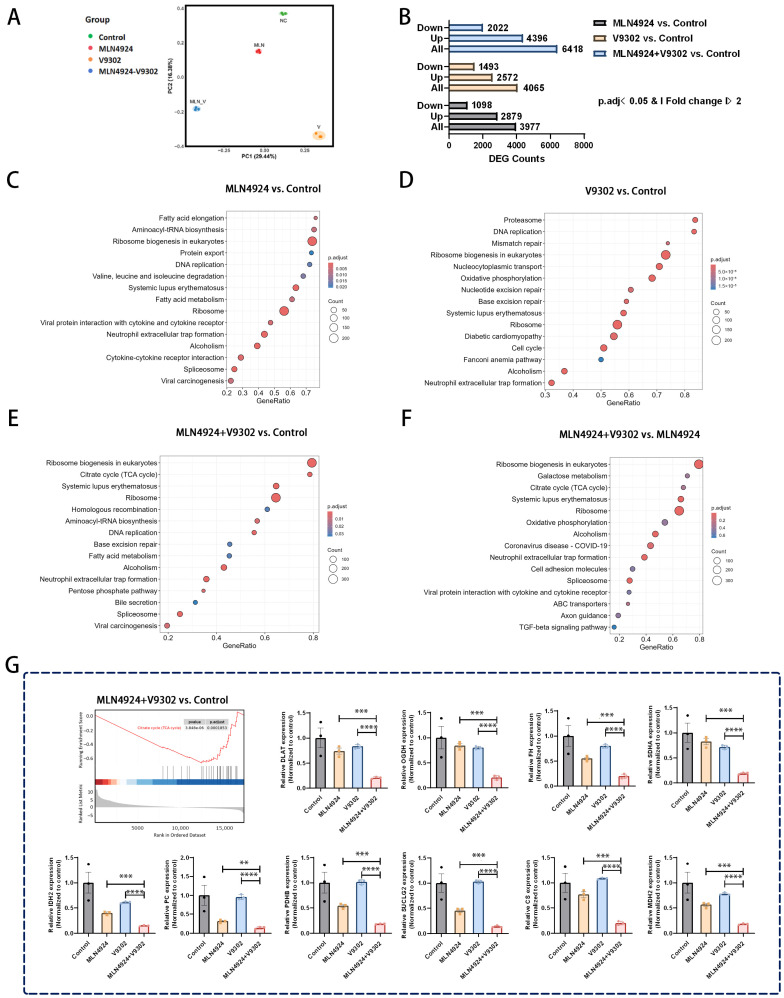
RNA-seq analysis revealed that MLN4924 in combination with V9302 inhibited the tricarboxylic acid (TCA) cycle. (**A**) PCA analysis revealed significant differences in gene expression profiles among groups. (**B**) Number of differently expressed genes (DEGs) in different treatment groups compared to control. (**C**–**F**) Gene Set Enrichment Analysis (GSEA) visualized the top 15 pathways (MLN4924 vs. control, V9302 vs. control, MLN4924 + V9302 vs. control, MLN4924 + V9302 vs. MLN4924). (**G**) GSEA enrichment analysis revealed that the “Citrate cycle (TCA cycle)” was significantly inhibited in the combination therapy group compared with the control group. Expression levels of key genes associated with the TCA cycle in different treatment groups. ** *p* < 0.01, *** *p* < 0.001, **** *p* < 0.0001.

To further assess potential molecular pathways of MLN4924 and V9302 to synergistically suppress AML, we performed transcriptome sequencing. There were significant differences in gene expression profiles among the four treatment groups [Figure 8A]. A total of 6418 differentially expressed genes (DEG) were identified between the MLN4924 + V9302 group and the control group, of which 4396 genes were upregulated and 2022 genes were downregulated [Figure 8B]. A total of 3977 DEGs were identified between the MLN4924 group and the control group, of which 2879 genes were upregulated and 1098 genes were downregulated [Figure 8B]. A total of 4065 DEGs were identified between the V9302 group and the control group, of which 2572 genes were upregulated and 1493 genes were downregulated [Figure 8B]. There were more DEGs in the MLN4924 + V9302 group than in the monotherapy group, suggesting that the combination of the two drugs affected more molecular pathways in AML cells. KEGG pathway enrichment analysis of these differential genes revealed that MLN4924 and V9302 alone significantly affected amino acid metabolism, DNA repair, and other pathways, while TCA cycle pathways were significantly enriched in the combination group [Figure 8C–F].

Gene Set Enrichment Analysis (GSEA) of these DEGs demonstrated that the Citrate cycle (TCA cycle) was significantly inhibited in the MLN4924 + V9302 group [Figure 8E–G]. Furthermore, we focused on changes in key genes associated with the TCA cycle, showing significant downregulation of many key genes in MLN4924 + V9302 compared to the monotherapy and control groups [Figure 8G]. The TCA cycle plays an important role in maintaining cell homeostasis. These results indicate that the combination of MLN4924 and V9302 significantly suppresses the TCA cycle in AML cells, potentially representing a key mechanism through which AML is suppressed.

## 4. Discussion

Acute myeloid leukemia (AML) is a highly heterogeneous hematological malignancy. With the continuous updating of treatment strategies, the prognosis of AML patients has improved significantly, but overall survival of AML remains a concern, and there is an urgent need to further explore effective strategies to prolong survival in AML patients [1].

In this study, we first explored MLN4924-regulated pathways through protein profiling. Protein profiling identified significantly upregulated GLUL after MLN4924 treatment. GLUL is involved in glutamine synthesis and significantly upregulated in glutamine deprivation [19,28]. Based on proteomic results and published studies, it is suggested that the significant upregulation of GLUL after MLN4924 treatment may be a signal of relative glutamine deficiency in AML cells. MLN4924 treatment may promote glutamine metabolism, resulting in increased glutamine consumption. AML cells enhance the endogenous glutamine synthesis pathway by upregulating GLUL after MLN4924 treatment. We further verified the expression of GLUL after MLN4924 treatment with different concentrations through RT-qPCR and Western blotting, and the results showed that GLUL was upregulated at both mRNA and protein levels after MLN4924 treatment. To further verify that the upregulation of GLUL after MLN4924 treatment is due to relative glutamine deficiency, we examined GLUL expression after increasing exogenous glutamine concentrations. The results showed that increasing glutamine concentrations in the medium (from 2 mM to 6 mM) downregulated GLUL expression, reversing the upregulation of GLUL by MLN4924. Importantly, we verified that MLN4924 promotes exogenous glutamine consumption by AML cells. These results suggest that MLN4924 treatment promotes glutamine metabolism in AML cells, resulting in relative intracellular glutamine deficiency. Combined with the above results and the glutamine-tropic properties of AML cells, we hypothesized that MLN4924 could protect AML cells from drug injury and promote survival by regulating glutamine metabolism.

Exogenous glutamine uptake pathways are also important for AML cell survival. Based on the above studies, we further focused on whether the exogenous glutamine uptake pathway was affected after MLN4924 treatment. We verified MLN4924 regulation of SLC1A5, a key transporter involved in glutamine uptake [29,30]. SLC1A5 is important for AML cell survival [31]. The results showed that MLN4924 treatment significantly upregulated SLC1A5 expression on the AML cell surface. These results further confirm that MLN4924 treatment promotes glutamine metabolism in AML cells and increases exogenous glutamine uptake. A study by Chen Y suggests a potential regulatory role of glutamine metabolism in AML progression [32]. MLN4924 treatment was shown to affect glutamine metabolism in breast cancer cells in a study by Zhou Q et al. [33]. Our results reveal that MLN4924 affects glutamine metabolism reprogramming in AML cells from different molecular perspectives.

The correlation between abnormal gene expression and the clinical prognosis of patients is key to becoming a therapeutic target. To further verify the potential clinical significance of GLUL and SLC1A5, we analyzed the expression of GLUL and SLC1A5 in AML patients using data from public databases and analyzed the correlation between their expression levels and the prognosis of AML patients. The results showed that GLUL and SLC1A5 were significantly upregulated in AML compared to hematopoietic stem cells from healthy donors. In addition, the expression levels of GLUL and SLC1A5 were associated with prognosis in AML patients; patients with high expression of GLUL had a worse prognosis, and, similarly, patients with high expression of SLC1A5 had a worse prognosis. Zhang W et al. showed that GLUL is associated with the prognosis of AML patients [34]. SLC1A5 was shown to play a critical role in AML cell survival in the study by Amaya ML et al. [35]. Combining our data with the published literature suggests that GLUL and SLC1A5 play an important role in AML.

In AML, MLN4924 treatment promotes glutamine metabolism, which may be a survival mechanism for AML cells against drug-induced cell damage. Inhibition of the glutamine metabolic pathway may be a potential strategy to further inhibit AML. Thus, we validated the effect of inhibiting GLUL and SLC1A5 on AML cells. We found that GLUL inhibition did not further increase AML cell sensitivity to MLN4924. We hypothesized that this may be due to the exogenous glutamine pathway compensating for the glutamine deficiency caused by inhibition of the endogenous glutamine pathway. Further, we focused on SLC1A5, a key protein involved in exogenous glutamine uptake. The results showed that knockdown of SLC1A5 could further increase AML cell sensitivity to MLN4924. Further, we verified the effects of MLN4924 and SLC1A5 inhibitor V9302 on AML cell proliferation, cell differentiation, mitochondrial membrane potential, and apoptosis. The results showed that MLN4924 combined with V9302 had synergistic anti-AML effects, significantly inhibited AML cell proliferation, and induced differentiation, mitochondrial membrane potential disorder, and apoptosis. In addition, MLN4924 in combination with V9302 also showed significant anti-leukemic effects in vivo. These results demonstrate the potential of targeting neddylation and glutamine metabolism in the treatment of AML. Notably, MLN4924 significantly upregulated SLC1A5 expression, and MLN4924 in combination with SLC1A5 inhibitor V9302 significantly induced AML differentiation. These results suggest a potential correlation between SLC1A5 expression and AML differentiation, and we will continue to investigate this direction in depth.

To further investigate the molecular mechanism underlying the synergistic anti-leukemic effects of MLN4924 and V9302, we conducted transcriptome sequencing. The results showed that the TCA cycle of AML cells treated with MLN4924 and V9302 was significantly inhibited. Several key genes associated with the TCA cycle were significantly downregulated in the combination group compared with the monotherapy group. TCA cycle plays an important role in maintaining cell homeostasis [36]. Inhibition of cycle components in the TCA cycle has been found to have anti-leukemic potential [37,38]. In our study, MLN4924 and V9302 co-treatment caused significant inhibition of the TCA cycle, revealing that MLN4924 and V9302 further led to metabolic disorders in AML cells, leading to apoptosis. Abnormal metabolic reprogramming is associated with leukemia genesis, progression, and drug resistance, and targeting metabolic pathways has become a potential anti-leukemia therapy strategy [39].

Our study demonstrates that MLN4924 treatment induces metabolic reprogramming in AML that promotes glutamine metabolism. This metabolic shift may represent a self-protective mechanism adopted by AML cells under therapeutic pressure. Therefore, we validate the anti-leukemia potential of MLN4924 in combination with targeting glutamine metabolism. The findings of this study further confirm the important role of metabolism in AML and provide some evidence for targeted metabolic strategies. However, there are still some questions that need to be further explored in this study. The relevant mechanisms involved in the regulation of glutamine metabolism reprogramming by MLN4924 need to be further explored in the future. In addition, the safety of MLN4924 in combination with V9302 was initially evaluated in this study, but more complete safety evaluation still needs to be verified in large samples.

## 5. Conclusions

In conclusion, our study reveals that MLN4924 treatment promotes glutamine metabolism and upregulates GLUL (a key enzyme involved in endogenous glutamine synthesis) and SLC1A5 (a key transporter regulating extracellular glutamine uptake) in AML cells. We found that inhibition of SLC1A5, but not GLUL, significantly enhanced AML cell sensitivity to MLN4924. In addition, we have also verified that co-targeting neddylation (MLN4924) and glutamine transporter SLC1A5 (V9302) can synergistically inhibit AML cells. The combination of MLN4924 and V9302 significantly inhibited AML cell proliferation, induced monocytic differentiation, and promoted apoptosis. Our study confirms the potential of targeted metabolic pathways in AML treatment. It is hoped that our study will provide new ideas for future treatment of AML.

## Data Availability

The data presented in this study are available upon request from the corresponding author.
